# Behavioral activation program for reducing depressive symptoms among the bereaved of cancer patients: A feasibility and preliminary effectiveness study in Japan

**DOI:** 10.1017/S1478951524001445

**Published:** 2024-11-07

**Authors:** Mariko Asai, Yuko Ogawa, Takatoshi Hirayama, Nozomi Sukigara, Eisho Yoshikawa, Sawako Furutani, Maiko Fujimori, Tatsuo Akechi, Shinichi Suzuki

**Affiliations:** 1 Department of Medical Psychology, Nippon Medical School, Tokyo, Japan; 2 Department of Student Counseling, Nippon Medical School, Tokyo, Japan; 3 Division of Supportive Care, Survivorship and Translational Research, National Cancer Center Institute for Cancer Control, Tokyo, Japan; 4 Graduate School of Clinical Psychology, Teikyo Heisei University, Tokyo, Japan; 5 Faculty of Pharma-Sciences, Teikyo University, Tokyo, Japan; 6 Psycho-Oncology Division, National Cancer Center Hospital, Tokyo, Japan; 7 Kokoro Support Clinic, Tokyo, Japan; 8 NPO Pancreatic Cancer Action Network, Chiba, Japan; 9 Department of Psychiatry and Cognitive-Behavioral Medicine, Nagoya City University Graduate School of Medical Sciences, Aichi, Japan; 10 Division of Palliative Care and Psycho-oncology, Nagoya City University Hospital, Aichi, Japan; 11 Faculty of Human Sciences, Waseda University, Saitama, Japan

**Keywords:** Behavioral activation, depression, bereaved, feasibility, preliminary effectiveness

## Abstract

**Objectives:**

This study aimed to examine the feasibility and preliminary effectiveness of a behavioral activation (BA) program for the bereaved of cancer patients toward reducing depressive symptoms.

**Methods:**

The BA program for the bereaved was a partially modified version for cancer patients. This program encompassed a preinterview and seven 50-minute sessions every 1–2 weeks, using worksheets, with homework assignments each day. To examine feasibility, the completion rates of intervention and 3 months of follow-up were examined. To examine the preliminary effectiveness, psychological symptoms were assessed with the Patient Health Questionnaire (PHQ-9; primary outcome) and Beck Depression Inventory-II (BDI-II) for depression and the Generalized Anxiety Disorder-7 (GAD-7) for anxiety. These were evaluated 3 times: before, immediately after, and 3-month post-intervention. Non-parametric tests were used for comparison of scores at 3 time points and calculation of effect size.

**Results:**

Of the 42 bereaved who were contacted, 21 were eligible and 20 were participated, while 19 and 18 were in the completed intervention and completed 3-month post-intervention categories (intervention completion rate was 95% and follow-up completion rate was 90%). PHQ-9, BDI-II, and GAD-7 showed significant reductions immediately and 3 months after the intervention compared to pre-intervention, and the effect sizes were all large after 3 months, although they were less than immediately after (PHQ-9: 0.89, 0.71; BDI-II: 0.88, 0.67; GAD-7: 0.57, 0.53).

**Significance of results:**

This study indicated that the BA program for the bereaved of cancer patients was feasible and effective vis-à-vis reducing depressive symptoms.

## Introduction

The bereavement of a loved one is a life event that dramatically changes the lives of bereaved families, with significant psychological or physical consequences for the bereaved. These include depression (Bradley 2004; Kris et al. 2004), prolonged grief disorder (Prigerson et al. 2009), physical illness such as cardiovascular disease (Carey et al. 2014; Prigerson et al. 1997), and suicide (Rostila et al. 2013). Interventions to reduce theses psychological or physical symptoms in bereaved families include psychoeducation on the dual process model of bereavement (Chow et al. 2019), group therapy based on meaning reconstruction theory (Chow et al. 2019), and Internet-based self-care program (Litz et al. 2014), and they have been reported to be effective, although the rationale and methods of intervention vary (Akechi et al. 2022).

Effective interventions have targeted only bereaved families with severe grief symptoms or used techniques such as behavior activation (BA) for depression (Eisma et al. 2015; Litz et al. 2014; Papa et al. 2013) or exposure to prolonged grief (Eisma et al. 2015; Litz et al. 2014; Shear et al. 2005) within cognitive behavior therapy. Notably, BA is a highly useful intervention that is as effective as other cognitive-behavioral therapies for depression (Lejuez et al. 2001), and it is inexpensive and easy to implement (Richards et al. 2016). When BA is applied to bereaved families, social withdrawal contributes to the complexity of grief, with the avoidance of associating with the deceased and ruminating on repeated reminders causing distress being associated with the onset and maintenance of grief. Therefore, BA is expected to encourage bereaved families to engage in activities perceived to be of value to them, thereby increasing behaviors that lead to positive moods and reducing grief (Eisma et al. 2015; Papa et al. 2013).

In cancer patients, BA has been reported to be effective in reducing depression in randomized controlled trials (Fernández-Rodríguez et al. 2021; Hopko et al. 2011). Notably, BA that contributes to reducing depression in cancer patients has been developed in Japan and reported to be feasible and potentially effective (Hirayama et al. 2023, 2019). In our survey of bereaved families in Japan, approximately half of the bereaved families who lost a family member to cancer had impaired mental health (Asai et al. 2013a), and the coping behaviors adopted by bereaved families were the most significant factor affecting their psychological state (Asai et al. 2012).

Based on the aforementioned, we hypothesize that the BA program for cancer patients (Hirayama et al. 2023), which has been reported to be effective in Japan, would also be effective in reducing depression in bereaved families of cancer patients. This study aims to examine the feasibility of a BA program for bereaved families of cancer patients and its preliminary effectiveness in reducing depressive symptoms.

## Methods

### Participants and procedure

A “Call for Participants” was posted on the website of the organizations to which the researchers belonged (Nippon Medical School and National Cancer Center Hospital). Moreover, bereaved families referred by the organizations whereto the researchers belonged (Teikyo Heisei University, Nippon Medical School, National Cancer Center Hospital, and Pan-Cancer Japan) were also eligible. Open calls for participants were carried out from October 2020 to December 2020 and from December 2021 to September 2023, for a total period of 25 months.

The inclusion criteria were the bereaved who (1) lost family members to cancer, (2) were aged 20 years or older, (3) were within 3 years of bereavement, (4) were with depressive symptoms (Patient Health Questionnaire [PHQ-9] of 10 or higher), (5) could speak Japanese, and (6) provided written consent to participate in this study. The exclusion criteria were the bereaved who (1) were with serious psychological symptoms (cognitive dysfunction, severe depression with suicidal ideation, etc.). For people aged 65 and over, the Mini-Mental State Examination was performed during the preliminary interview, and scores below 23 were excluded as cognitively impaired (Ideno et al. 2012); (2) had received psychological intervention by mental health professionals before enrollment; and (3) were deemed to have difficulty in participating in this program by the researcher due to other reasons. Participants were given a pre-paid card worth 1,000 yen per session as compensation. Assessment measures were administered pre-intervention (T1), post-intervention (T2), and 3-month post-intervention (T3).

This study was conducted in compliance with the Declaration of Helsinki and ethical guidelines for epidemiological research and was approved by the Institutional Review Board and Ethics Committee of the Teikyo Heisei University (R02-026) and Nippon Medical School (M-2021-004).

### Sample size

The number of people required to detect preliminary efficacy was calculated as follows: As there were no previous studies with bereaved families using the primary endpoint, the PHQ-9, at the time of study design, we referred to a randomized controlled trial using the secondary endpoint, the Beck Depression Inventory (BDI) (Litz et al. 2014), and calculated the required sample size thus: difference between group means of 8, standard deviation of 8, significance level of 5%, power of 80%, and drop-out rate of 20%. The total number of expected study subjects was set at 20.

### Intervention (BA program for the bereaved)

It is noteworthy that BA focuses on the context of life situations that cause the inhibition of activities and avoidance due to aversive experiences, breaks the vicious cycle, promotes and reinforces behaviors that are in line with participants’ original goals, and helps patients reaffirm the relationship between behavior and situation so that their own behaviors lead to the originally desired outcome.

Table 1 shows the BA program for the bereaved. It was a partially modified BA program for cancer patients (Hirayama et al. 2023, 2019) developed by researchers in this study (MA, YO, TH, and SS). This program entailed a pre-interview and seven 50-minute sessions with worksheets, and these were conducted every 1–2 weeks, with an average of 10 minutes of homework per day. Participants choose how to conduct all sessions, either face-to-face (at Nippon Medical School or National Cancer Center Hospital) or online (Webex or Zoom Corp.), before the commencement of the session, and this mode cannot be changed during the session. In online sessions, the worksheet file was shared on a computer screen by the therapist, the bereaved persons’ responses were filled in, and a printed copy was mailed after completion.

**Table 1.** Behavioral activation program for the bereaved of cancer patients

Session	Theme	Contents	Homework
	Pre-interviews	**Review on the experience of caring for cancer patients, end-of-life care, and post-bereavement**	List the following 2 points: (1) What is troubling you at the moment, and what makes it hard for you to think about it? (2) What kind of life do you want to lead and what do you hope the program will do for you?
1	Let’s begin	Understand the relationship between emotions and behaviors Learn tips on how to avoid being preoccupied by bereavement to regain pleasure and meaning in life	Daily record of behaviors and emotions
2	Identify the relationships between emotions and behaviors	Explore the relationship between emotions and daily activities Identify patterns of feeling negative and positive emotions	Daily record of behaviors and emotions
3	Identify activities that make your life pleasurable	Clarify life values Identify activities that are achievable in one’s current situation	Record of new behaviors
4	Review the results of activities	Evaluate the usefulness and difficulty of each activity Discuss ways to participate in challenging activities	Record of new behaviors
5	Identify difficult situations and understand ways to change one’s feelings	**Clarify the difference between ruminating and yearning. Both are memories of the deceased that suddenly come to mind, but he distinguished between ruminating, which leads to anxiety and depression, and yearning, which leads to calm** Identify situations that are likely to lead to anxiety and depression Identify thoughts that occur in the early stages of anxiety and depression as well as any vicious cycles that might be involved Identify pros and cons of negative thinking Ruminating situations and dealing with them Analysis of the advantages and disadvantages of ruminating or not ruminating	Record of new behaviors
6	Learn to live a life of value	Review how the program helped change patterns of daily life Identify future goals and make plans to achieve them	Daily record of behaviors and emotions
7	Wrap-up and graduation	Review what has been learned through the program Identify future issues and how to address them	

Note: Bold type indicates areas that have been modified for the bereaved.

In this BA program for the bereaved, there were 2 main modifications for the bereaved: first, pre-interviews were added before first session, and second, a self-analysis of ruminations was conducted during the fifth session. Pre-interviews were added to fully understand the bereaved family’s feelings, including regrets from pre-bereavement cancer care, to not only form a therapeutic alliance with the therapist but also motivate them to participate in the program. In addition, if possible, the pre-interviews were include to identify behaviors and thoughts that were avoided after bereavement because they were painful regarding remembering the deceased. In the fifth session, the therapist explained the difference between ruminating and yearning: “Both are memories of the deceased that suddenly come to mind,” but he differentiated between ruminating, which leads to distress, and yearning, which leads to calm. Then, self-analysis of ruminations was conducted. This was added because the authors’ previous study showed that the coping pattern of “Continuing Bonds Focused” was associated with impaired mental health (Asai et al. 2013a), and it was assumed that they used a lot of ruminations.

There were 2 types of homework: one was to record daily activities and the other was to record new behavior. Activity scheduling is a behavioral treatment for depression in which patients observe their mood and daily activities, increase enjoyable activities, and learn how to adjust their environment (Cuijpers et al. 2007).

The therapists were 2 psychotherapists (MA, YO) and 1 psychiatrist (TH) with sufficient clinical cancer experience. Two of them (YO, TH) were implementers of the BA program for cancer patients. The other (MA) was a psychotherapist with experience in caring for bereaved cancer patients, who had observed a BA program for cancer patients conducted by a psychiatrist (TH) and received guidance from a BA supervisor (SS) with sufficient knowledge and experience in BA, using a video of a BA program for the bereaved conducted for a simulated bereaved family.

Note that this study did not restrict the use of pharmacotherapy for the benefit of participants, as it has been reported that pharmacotherapy used in conjunction with psychotherapy is more effective in reducing depression than each alone (Wiles et al. 2013).

### Measures

#### Psychological symptom scale

##### PHQ-9: Primary outcome

PHQ-9 is a diagnostic tool for depression and other psychiatric disorders commonly seen in primary care. It is a self-administered questionnaire consisting of 9 items and assesses the frequency of 9 common symptoms of depression, including mood, sleep, fatigue, and appetite, on a 4-point scale (0–3) over the past 2 weeks. A score of 10 or higher is considered an indication of depression (Spitzer et al. 1999), and its reliability and validity have been verified in the Japanese version (Muramatsu 2014).

##### Beck Depression Inventory (BDI-II)

BDI-II is a self-administered questionnaire consisting of 21 items to determine the severity of depressive symptoms. Scores are recorded on a 4-point scale (0–3) over the past 2 weeks, with severity ranging from 0 to 13 for extremely mild disease, 14 to 19 for mild disease, 20 to 28 for moderate disease, and 29 to 63 for severe disease (Beck et al. 1996). In cancer patients, a score of 16 has been shown to be the cutoff for screening for depression (Warmenhoven et al. 2012).The validity of the Japanese version has also been confirmed (Kojima et al. 2002).

##### Generalized Anxiety Disorder (GAD-7)

The GAD-7 is a self-administered questionnaire consisting of 7 items to provide a brief assessment of generalized anxiety disorder. The scale asks for a score on a 4-point scale (0–3) for the past 2 weeks. The total score ranges from 0 to 21, with 0 to 4 indicating no generalized anxiety disorder, 5 to 9 mild, 10 to 14 moderate, and 15 to 21 severe (Spitzer et al. 2006). The validity of the Japanese version has also been confirmed (Muramatsu et al. 2010).

##### Associated factors scale for psychological symptoms

The following were selected as intervention effects and relevant factors for psychological symptoms: activation and avoidance (BADS-SF) as targeted behaviors of BA, and value (VQ) and reward (RPI) as cognitions. These were assessed, in addition to our developed coping behavior after bereavement (CSB-SF) as intervention effect factors.

##### Behavioral Activation for Depression Scale-Short Form (BADS-SF)

The BADS-SF is a shortened version of the Behavioral Activation for Depression Scale (BADS) developed to assess behavioral changes resulting from BA. This is evaluated on a 7-point scale (0–6) designed to measure 2-factor “activation” and “avoidance” related to BA (Manos et al. 2011). The Japanese version consists of 8 items, 1 less than in the original version, and the validity of the Japanese version has also been confirmed (Yamamoto et al. 2015).

##### Coping Strategies after Bereavement-Short Form (CSB-SF)

The CSB-SF is a shortened version of the Coping Strategies after Bereavement scale (CSB), a 38-item 5-point scale (0–4) designed to assess post-bereavement behavior: distraction, continuing bonds, and social sharing/reconstruction (Asai et al. 2013a). Based on the results of factor analysis (Asai et al. 2012), the 3 items with the highest factor loadings for each subscale were selected for a total of 9 items. Three items of each subscale are: for distraction, “do physical exercise,” “play my role in daily life,” and “go out for change of pace”; for continuing bonds, “look back on memories of the deceased,” “have inner conversations with the deceased,” and “talk about the deceased”; and for social sharing/reconstruction, “make my bereavement experience contribute to others,” “seek for support from the bereaved,” and “make contribution to social activity with the cherished desire of the deceased.”

##### Valuing Questionnaire (VQ)

VQ is a self-administered questionnaire consisting of 10 items on a 7-point scale (0–6) that measures the consistency of one’s behaviors with their values (Smout et al. 2014). This scale is designed to measure 2 factors, namely, progress and obstruction, and the Japanese version has been confirmed to be sufficiently reliable and valid (Doi et al. 2017).

##### Reward Probability Index (RPI)

RPI measures the degree of perceived environmental reward (Carvalho et al. 2011). The Japanese version of the RPI consists of 19 items on a 4-point scale (1–4), 1 fewer than the original scale, including 3 factors: amount of reward, environmental suppressors, and reward skill. The reliability and validity of the Japanese version have been confirmed (Yamamoto et al. 2016).

### Statistical analysis

#### Feasibility

An intervention completion rate of 80% or higher is considered “excellent feasibility,” and 60–80% is considered “good feasibility.” If the percentage falls below 60% during the course of the project, it will be suspended.

#### Preliminary effectiveness

The results of testing the normal distribution by Shapiro–Wilk for all measures included scores that did not show a normal distribution, so non-parametric tests were used for all statistical analyses.

Friedman repeated-measures analysis of variance on ranks with Bonferroni correction was used to compare of scores at 3 time points: T1, T2, and T3. The effect size of the intervention (*r*) was calculated by dividing the test quantity standardized to a *z*-value via the Wilcoxon signed-rank test by the square root of the number of participants. Effect sizes of 0.1, 0.3, and 0.5 indicate small, medium, and large, respectively (Cohen 1992). To examine factors associated with psychological symptom, Spearman’s *ρ* was calculated to examine correlations of nonparametric variables. A significant difference was defined as a *p*-value of <0.05. Data were analyzed with the SPSS version 29.0 (IBM Corp.).

## Results

### Participants’ characteristics

The demographic characteristics of 20 participants of this study are presented in Table 2. Notably, 17 females and 3 males with a median age of 55 years were included. The post-bereavement period ranged from 2 to 30 months, with a median of 6.5 months. Nine used the face-to-face mode, while 11 used the online intervention method.

**Table 2.** Participant’s characteristics (*n* = 20)

	Mean ± SD (median, range)	*n* (%)
Age, years	55 ± 8.8 (55, 34–72)	
Time since bereavement, months	12 ± 9.5 (6.5, 2–30)	
Gender		
Male		3 (15)
Female		17 (85)
Relationship of the deceased		
Spouse		13 (65)
Parent		2 (10)
Child		3 (15)
Siblings		2 (10)
Living status		
Living alone		11 (55)
Living with someone		9 (45)
Employment status		
Social sharing/reconstruction		11 (55)
Unemployed		6 (30)
Other		3 (15)
Present disease		
Presence		14 (70)
Absence		6 (30)
Deceased’s cancer cite		
Pancreas		9 (45)
Lung		2 (10)
Brain		2 (10)
Other		7 (35)
Intervention method		
Face-to face		9 (45)
Online		11 (55)

SD = standard deviation.

### Feasibility

**Figure 1.**
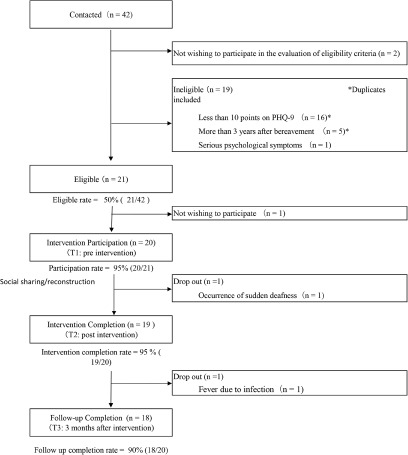
Flow of study sample.

Figure 1 shows the flow of the study sample. Of the 42 bereaved who were contacted, only 6 bereaved contacted us on their own without being referred by the researcher after seeing the call for research participants posted on the website; most of the rest were referred by the researcher. Moreover, 21 were eligible, the eligibility rate was low at 50%, and most of the ineligible patients had PHQ-9 scores of less than 10 (16/21). Furthermore, 20 participated (participation rate, 95%), 19 belonged to the completed intervention group (intervention completion rate, 95%), and 18 were in the 3-month post-intervention category (follow-up completion rate, 90%). The reason for dropout was sudden deafness in one after participation and fever due to infection in one during follow up.

### Preliminary effectiveness

Table 3 shows the preliminary effectiveness of the BA program for the bereaved. The psychological symptom rating scales – PHQ-9, BDI-II, and GAD-7 – all showed significant reductions immediately and 3 months after the intervention compared to pre-intervention, and the effect sizes were all large after 3 months, although they were less than immediately after (PHQ-9: 0.89, 0.71; BDI-II: 0.88, 0.67; GAD-7: 0.57, 0.53). Significant changes with respect to the associated factor rating scale were: increased BADS-SF activation immediately after, decreased BADS-SF avoidance after 3-month post-intervention, increased CSB-SF distraction, and increased RPI amount of reward.

**Table 3.** Comparison of scores at 3 time points (T1, T2, T3) and effect size of intervention

	Score range	Pre-intervention (T1)		Post-intervention (T2)		3-Month post-intervention (T3)		Comparison of scores at 3 time points		Effect size (*r*)^b^	
								Friedman’s rank test	Multiple comparisons	Wilcoxon signed-rank test	
		Median	Average rank	Median	Average rank	Median	Average rank	*p* value	*p* value < 0.05	Pre–post (T1–T2)	Pre–3M (T1–T3)
PHQ-9	0–27	14.5	2.78	9.00	1.36	7.50	1.86	<0.01	T2, T3 < T1	0.89	0.71
BDI-II	0–63	32.0	2.79	12.0	1.44	10.0	1.76	<0.01	T2, T3 < T1	0.88	0.67
GAD-7	0–21	9.00	2.64	3.00	1.72	2.00	1.64	<0.01	T2, T3 < T1	0.57	0.53
BADS-SF											
Activation	0–30	7.00	1.50	12.0	2.41	13.0	2.09	0.03	T1 < T2	0.65	0.51
Avoidance^a^	0–18	9.50	1.56	12.0	2.06	12.5	2.39	0.03	T1 < T3	0.43	0.38
Total	0–48	17.0	1.56	23.5	2.24	24.0	2.21	0.08		0.63	0.47
CSB-SF											
Distraction	0–12	7.00	1.56	8.50	1.97	9.50	2.47	0.01	T1 < T3	0.55	0.62
Continuing bonds	0–12	9.00	1.83	9.50	2.14	9.00	2.03	0.55		0.25	0.07
Social sharing/reconstruction	0–12	4.50	1.94	7.00	2.11	6.00	1.94	0.83		0.13	0.14
Total	0–36	21.00	1.72	22.5	2.19	25.5	2.08	0.32		0.38	0.4
VQ											
Progress	0–30	9.50	1.72	14.0	2.03	15.5	2.25	0.25		0.34	0.33
Obstruction^a^	0–30	11.5	1.69	16.0	2.03	16.5	2.28	0.19		0.38	0.42
Total	0–60	22.5	1.89	28.5	2.11	32.0	2.00	0.80		0.33	0.42
RPI											
Amount of reward	7–28	14.0	1.47	17.0	2.18	17.5	2.35	0.02	T1 < T3	0.50	0.54
Environmental suppressors^a^	9–36	23.0	1.59	26.0	2.19	26.0	2.22	0.10		0.46	0.52
Reward skill	3–12	6.00	1.88	6.00	2.00	6.00	2.12	0.72		0.08	0.15
Total	19–76	41.5	1.59	50.0	2.13	50.5	2.28	0.11		0.36	0.59

PHQ = Patient Health Questionnaire; BDI = Beck Depression Inventory; GAD = Generalized Anxiety Disorder; BADS-SF = Behavioral Activation for Depression Scale-Short Form; VQ = Valuing Questionnaire; RPI = Reward Probability Index; CSB-SF = Coping Strategies after Bereavement-Short Form.

a Reverse scale items: Higher scores indicate lower avoidance, obstruction and environmental suppressors.

b Effect size (*r*): small 0.10, medium 0.30, large: 0.5.

Negative correlations with PHQ-9 scores immediately after the intervention were large and significant for both subscales and total scores of the associated factors scales (Spearman’s *ρ* = −0.50–0.81, *p* < 0.05), but only “continuing bonds” was small and not significantly different (Spearman’s *ρ* = −0.27, *p* = 0.29).

A preliminary study was conducted to determine whether the immediate post-intervention effect size on PHQ-9 differed by participants characteristics (divided into 2 groups): age (10 under 55 years, 10 over 55 years), duration since bereavement (13 under 1 year, 7 over 1 year), relationship (13 spouses, 7 others), cohabitant (11 without, 9 with), occupation (9 without, 11 with), method (9 face-to-face, 11 online), and pre-intervention PHQ-9 scores (10 with 10–14 points, 10 with 15 or more points). Immediately after the intervention, both effect sizes were large (0.88–0.90), and there were no significant differences between the 2 groups.

## Discussion

This is the first study to examine the feasibility and preliminary effectiveness of a BA program for reducing depressive symptoms among the bereaved of cancer patients.

First, with regard to feasibility, the intervention completion rate was 95% (19/20), and the 3-month follow-up completion rate was 90% (18/20). The intervention completion rate was more than 80%, typifying “excellent feasibility” according to the criteria developed at the time of study design. This rate was higher than in previous studies with cancer patients (75% and 69%, respectively) (Hirayama et al. 2023), indicating that a BA program targeting bereaved families was fully feasible. The only reason for dropout was physical symptoms. Owing to the high drop-out rate of BA in the previous Internet intervention (Eisma et al. 2015), the present study set up a pre-interview, and it emphasized relationship building with the therapist, which was effective considering that grief was an emotional dysfunction that involved interpersonal anger due to the loss of the deceased (Eisma and Stroebe 2021). Furthermore, the fact that the intervention could be conducted online as well as in person could help to further disseminate it.

Second, regarding preliminary effectiveness, the psychological symptom rating scales, namely, PHQ-9, BDI-II, and GAD-7, all showed significant reductions immediately and 3 months after the intervention compared to pre-intervention, and both showed large effect sizes immediately and 3 months after the intervention, suggesting that the BA program was effective for depression and anxiety. Behavioral (increased BA, decreased avoidance, and increased distraction) and cognitive (increased reward) aspects were also observed as results, thereby indicating that BA had an impact on the reduction of these symptoms. Furthermore, the PHQ-9, which can assess depression with 9 items, was used in this study, and its effect size was comparable (0.89, 0.88) to the 21-item BDI-II, indicating that it could replace it as a simple depression rating scale. We reviewed papers up to 2020 and found that the effect size (standardized mean difference) of psychosocial interventions for the bereaved on depression was −0.56, which was moderate (Akechi et al. 2022). Although the results of this study cannot be compared with those of the present study only numerically, the BA program’s effect size on depression was 0.89 immediately after and 0.71 after 3-month post-intervention, both of which indicated a large effect size, and future research in connection to the effectiveness of the program is expected. There was no program in Japan that had been demonstrated through randomized controlled trials to reduce depression in bereaved families, and as the BA program used in this study is also available online, it is expected to be widely used by bereaved families in need of intervention.

Additionally, 2 preliminary findings are presented as follows: First, the negative correlations with PHQ-9 scores immediately after the intervention were large and significant for both subscales and total scores on the related factor scale, suggesting that improvements through BA in terms of both behavioral and cognitive aspects influenced the reduction of depressive symptoms. Only “continuing bonding” was not significantly associated with PHQ-9 scores immediately after the intervention, suggesting that it was not associated with depressive symptoms and that other post-bereavement coping behaviors such as distraction and social sharing/restructuring increased, which might contribute to the improvement of depressive symptoms. Second, a preliminary examination of whether the immediate post-intervention effect sizes on PHQ differed by attribute showed that they were equivalent for age, length of time since bereavement, relationship, and so on. In particular, no differences were found between the face-to-face and online modes, suggesting that online delivery might be sufficient in the future. However, these sub-analyses are small, with only about 10 participants, and they are only preliminary studies.

Although this study reported a bereavement program that was feasible and effective in reducing depression, significant challenges were also observed regarding the improvement of bereavement care in Japan. The results of this study indicated that only a small percentage of bereaved families voluntarily participated in the study, and that the assessment of the severity of depression did not agree among the bereaved families themselves, the referrer’s assessment, and the scale assessment. Therefore, a support system that can recruit and assess symptoms is needed to provide appropriate care to bereaved families who need care. In Japan, approximately 390,000 people die from cancer each year (Ministry of Health, Labour & Welfare of Japan 2022), and although about half of them have psychiatric symptoms (Asai et al. 2013a), medical personnel have little opportunity to come into contact with bereaved families. Therefore, it is important to collaborate with people in the local community for the social implementation of a support system for bereaved families, and how, when, and with whom is an issue to be addressed further in the future.

Some limitations of this study are stated as follows: First, there was sample bias in participants. Put differently, it is possible that the small number of bereaved families who have participated without the researcher and their high expectations of the program may have influenced completion rates and effectiveness. Second, this study did not restrict the use of pharmacotherapy for the benefit of participants, so the few who used them might have influenced the effectiveness of this study. Third, this study did not use measures of grief or Post Traumatic Stress Disorder (PTSD), which are symptoms specific to bereaved families. This was influenced by the fact that it was a time when psychiatric diagnoses such as Diagnostic and Statistical Manual of Mental Disorders (DSM) and International Classification of Diseases (ICD) were being revised with regard to these traumatic symptoms. Fourth, study design is a before-and-after comparative study and does not set up a control group, which contains bias with regard to effectiveness. Moreover, the small number of subjects and the non-normative nature of the data means that the study is not comparable to previous studies in terms of effectiveness. Further studies with randomized controlled trials are needed to determine efficacy. Fifth, the quality of the intervention was not adequately controlled, although the program content was the same because the intervention was conducted with 3 therapists, both face-to-face and online, and it took approximately 2 years for recruitment. Although these were all unavoidable at a time when COVID-19 infection control measures were required, there is room for improvement.

In conclusion, this study indicated that a BA program for the bereaved of cancer patients was feasible and effective for reducing depressive symptoms.
